# Merging Orthovoltage X-Ray Minibeams spare the proximal tissues while producing a solid beam at the target

**DOI:** 10.1038/s41598-018-37733-x

**Published:** 2019-02-04

**Authors:** F. Avraham Dilmanian, Sunil Krishnan, William E. McLaughlin, Brendan Lukaniec, Jameson T. Baker, Sandeep Ailawadi, Kara N. Hirsch, Renee F. Cattell, Rahul Roy, Joel Helfer, Kurt Kruger, Karl Spuhler, Yulun He, Ramesh Tailor, April Vassantachart, Dakota C. Heaney, Pat Zanzonico, Matthias K. Gobbert, Jonathan S. Graf, Jessica R. Nassimi, Nasrin N. Fatemi, Mark E. Schweitzer, Lev Bangiyev, John G. Eley

**Affiliations:** 1 Department of Radiology, Stony Brook University Hospital, Stony Brook, NY 11794 USA; 2 Department of Radiation Oncology, Stony Brook University Hospital, Stony Brook, NY 11794 USA; 3 Department of Neurology, Stony Brook University Hospital, Stony Brook, NY 11794 USA; 4 Department of Psychiatry, Stony Brook University Hospital, Stony Brook, NY 11794 USA; 5 Department of Biomedical Engineering, Stony Brook University Hospital, Stony Brook, NY 11794 USA; 6 Stony Brook Cancer Center, Stony Brook University Hospital, Stony Brook, NY 11794 USA; 7 Department of Radiation Oncology, University of Texas MD Anderson Cancer Center, Houston, TX 77030 USA; 8 Precision X-ray Inc., North Branford, CT 06471 USA; 9 Department of Radiation Medicine, Northwell Health Medical Center, Northwell, NY USA; 10 Loma Linda University School of Medicine, Loma Linda, CA USA; 11 Memorial Sloan Kettering Cancer Center, New York, NY USA; 12 Department of Mathematics and Statistics, University of Maryland, Baltimore County, Baltimore, MD 21250 USA; 13 Department of Radiology, City of Hope, Duarte, CA 91010 USA; 14 Department of Radiation Oncology, University of Maryland School of Medicine, Baltimore, MD 21201 USA

## Abstract

Conventional radiation therapy of brain tumors often produces cognitive deficits, particularly in children. We investigated the potential efficacy of merging Orthovoltage X-ray Minibeams (OXM). It segments the beam into an array of parallel, thin (~0.3 mm), planar beams, called minibeams, which are known from synchrotron x-ray experiments to spare tissues. Furthermore, the slight divergence of the OXM array make the individual minibeams gradually broaden, thus merging with their neighbors at a given tissue depth to produce a solid beam. In this way the proximal tissues, including the cerebral cortex, can be spared. Here we present experimental results with radiochromic films to characterize the method’s dosimetry. Furthermore, we present our Monte Carlo simulation results for physical absorbed dose, and a first-order biologic model to predict tissue tolerance. In particular, a 220-kVp orthovoltage beam provides a 5-fold sharper lateral penumbra than a 6-MV x-ray beam. The method can be implemented in arc-scan, which may include volumetric-modulated arc therapy (VMAT). Finally, OXM’s low beam energy makes it ideal for tumor-dose enhancement with contrast agents such as iodine or gold nanoparticles, and its low cost, portability, and small room-shielding requirements make it ideal for use in the low-and-middle-income countries.

## Introduction

Nearly all patients undergoing brain tumor radiation therapy, and those undergoing radiosurgery for the treatment of focal epilepsy, develop a certain level of cognitive deficits long-term^1^. The effect is particularly severe in children^2–4^. In this regard, the cortex plays a major role in the mediation of the long term radiation damage to the brain. This is because of the integral role that the cortex plays with hosting the gliogenesis process where the fate of the neural stem cells (NSC), in terms of their differentiation into glial cells, is determined^3^. The physical basis for the damage to the non-targeted tissues from MV x-rays or Co-60 gamma rays is the spatial distribution of the radiation they produce in the brain. Specifically, the doses produced to the brain tissue located proximal and distal to the target are excessive.

**Figure 1:**
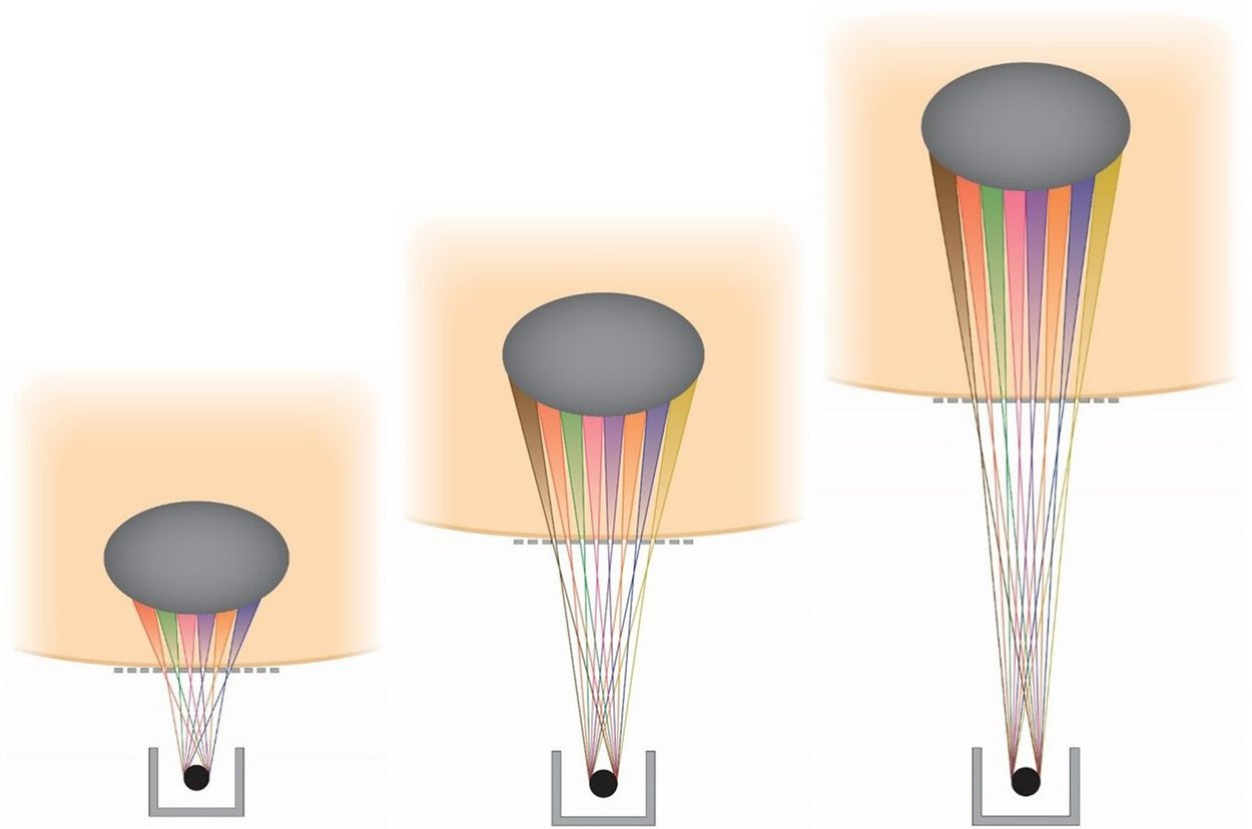
Schematic view of the method Orthovoltage x-ray minibeams (OXM). The figure also demonstrates the method of adjusting the tissue depth at which the minibeams merge by varying the distance between the source and the multislit collimator.

The method we are presenting, merging Orthovoltage X-ray Minibeams (OXM) (Fig. 1), uses the very large tissue-sparing effect of x-ray minibeams^5^ that was established with synchrotron x-rays. Specifically, the study reported in ref.^5^ indicates that irradiation of the entire rat brain with 0.68-mm minibeams with 1.36-mm beam spacing on-center at 170 Gy was tolerated for the 7 months of follow-up with no functional disturbance except for a two-hour transient effect shortly after the irradiation^5^. The x-ray minibeams are produced by positioning a multi-slit collimator in the path of the incident beam near the subject’s skin. Furthermore, it produces a solid beam at the target where the minibeams merge with their neighbors due to the gradual broadening of such minibeams. This beam broadening is produced by the large source spot size (3–9 mm) of the machines and by the relatively small source-to-collimator distance, 10–30 cm to be used in a clinical geometry (Fig. 1). In this way, the skin and the entire amount of tissue proximal to the target can be spared because they are exposed only to minibeams, while the target is exposed to a therapeutic solid-beam dose. Another major physical attribute of OXM is its extremely sharp lateral dose falloff compared to that of MV x-rays. The physical basis for the effect is that orthovoltage x-rays interact with tissues to a large extent by photoelectric interaction, while the mode of interaction of MV x-rays with tissues is nearly completely Compton scattering, which produces highly non-local dose around the point of interaction.

**Figure 2:**
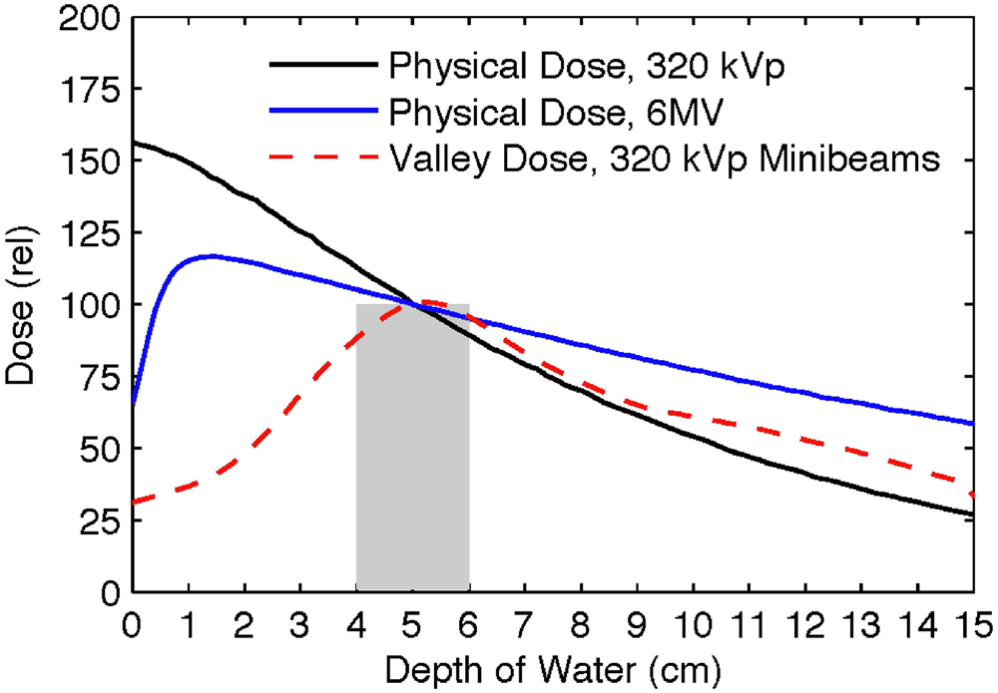
Monte Carlo simulations comparing depth-dose distributions of 6-MV and 320-kVp x-rays with the incident valley dose curve for the 320-kVp OXM.

Orthovoltage x-ray therapy was the only method of external-beam radiation therapy for the first half of the 20^th^ century until it was replaced by megavolt x-rays, produced by electron linacs, for deeper dose penetration to tissues. Figure 2 shows the depth-dose distributions of three different beams in a 15-cm water phantom. These are (a) a measured 6-MV x-ray beam (Varian TrueBeam accelerator, 10 × 10 cm^2^ field size, 100-cm source-to-surface distance (SSD), (b) a simulated 320-kVp orthovoltage x-ray beam with 3.8-mm Cu half-value layer (10 × 10 cm^2^ field size, 50-cm SSD), and (c) the predicted valley dose curve for an x-ray microbeam array of 0.3 mm minibeams spaced 0.7 mm on center segmented from the second curve, that is elaborated on in the Results. The orthovoltage simulations were carried out by MCNPX version 2.7 (Los Alamos National Laboratory, Los Alamos, NM) using the rough assumption that the biological dose was the same as the “valley dose” produced by the minibeam array in the subject. The significance of this assumption is that it assumes that the “peak” minibeams dose has no significance in determining the radiation damage to the tissue; rather it is only the valley dose that determines the extent of the biologic effect. This is because at high doses the valley dose acts as a solid beam above which the peaked minibeams ride. The shape of the resulting depth-dose curve presenting the minibeams’ valley dose in Fig. 2 indicates that the tissue-sparing of minibeams can reach the depth of 5 cm or larger in the subject, which would indicate that the cerebral cortex can be spared. The three curves in that figure were normalized to each other to coincide at the center of a 2-cm target at the depth of 5 cm for dose comparison between OXM and MV x-rays in the proximal and distal sides of the target.

The tissue-sparing effect of arrays of parallel, thin x-rays, commonly called microbeams for beams narrower than ~100 µm and minibeams for 100 to 700-µm beams, has been established over about 30 years with synchrotron-generated x-rays^5–12^. The first such study was carried out at the National Synchrotron Light Source (NSLS), Brookhaven National Laboratory (BNL) in the early 1990s^6^. In that study, rats irradiated in nearly their entire cerebellum with an array of 37-µm x-ray microbeams spaced 75-µm on-center at 250 Gy in-beam in-depth cerebellar dose (produced by 1,000 Gy entrance dose from the front of the head) did not show any adverse effect during a three month evaluation, and no tissue damage in the cerebellum was observed in their H&E-stained brain tissue at that time^6,7^. The study produced much excitement and led to the performance of a large number of microbeam studies on several central-nervous system (CNS) models at both the NSLS^5–8,10,12,13^ and the European Synchrotron Radiation Laboratory (ESRF)^9,11,14–16^. Approximately 15 years later, it was shown again at the NSLS that microbeams as thick as 0.68 mm (called minibeams here for beams of 0.3 mm or larger thickness) still retained much of their tissue-sparing effect in the rat spinal cord and brain^5^. In particular, as indicated above, irradiation of the entire rat brain with 0.68-mm minibeams with 1.36-mm beam spacing on-center at 170 Gy was tolerated remarkably well.

## Significance of the results presented in Fig. 2 for estimating the potential therapeutic efficacy of OXM

The comparison of the OXM’s valley dose curve to the two other curves in Fig. 2 points to two significant phenomena. First, the incident dose, i.e., the predicted biologic skin dose from the minibeams’ array, is substantially smaller than that of the 6-MV beam. This is remarkable considering the significance that is commonly attributed in the field to the MVs’ “skin-sparing” effect. The second phenomenon, which is by far more significant than the first one, is that the proximal tissue-sparing effect of the x-ray minibeams can persist over several centimeters of tissue depth, e.g., ~3-cm in the example of Fig. 2. This depth of tissue-sparing, which can be much more than 3 cm in clinical situations, is sufficiently large to allow sparing of the entire cerebral cortex when viewed from all directions around the head. Considering the very large radiosensitivity of the cerebral cortex, as elaborated on in the discussion section, this means that OXM can be used to treat brain tumors with nearly complete sparing of the cerebral cortex. This would be a major effect not only for the pediatric patients for whom the well-being of the cerebral cortex is much more vital, but also for adults.

## Results

The results presented below in Parts A and B obtained under the experimental methodologies substantiate the basic concepts of orthovoltage x-ray minibeams. These include (a) to produce more evidence on the tissue sparing effect of the x-ray minibeams, particularly when very high doses are used, and (b) to provide experimental evidence for the method’s flexibility to adjust the beam merging depth in the tissue at the desired value. For this purpose, we define two terms, a physical one and a biological one. First, the physical term “beam merging depth” is quantitatively defined as the tissue depth at which the peak-to-valley ratio is 1.2:1.0 or smaller. Second, the biological term “tissue-sparing” is defined to be when the peak-to-valley ratio is 3:1 or larger. Of course, the beam’s attenuation rate with subject depth is also of major importance because it signifies the prospect for the clinical use of the method.

We note that in the following discussion, the term “dose” means physical absorbed dose, unless otherwise indicated.

### Part A. Measured Results

#### A1. Cross Sectional Measurements

**Figure 3:**
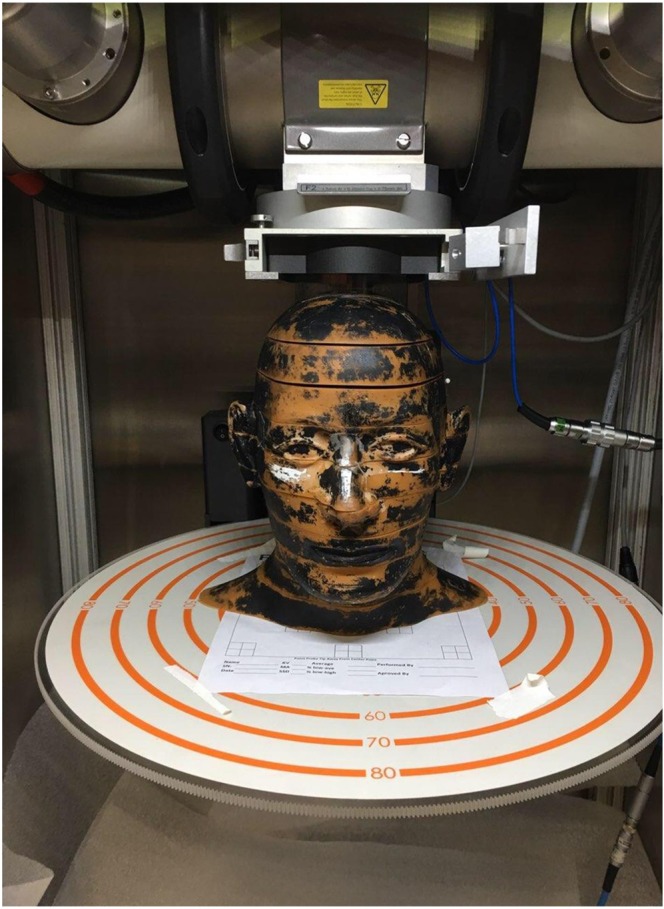
Alderson phantom positioned underneath the beam-defining collimator of the 250 kVp x-ray machine at PXI. The multislit collimator, which was used to produce Fig. 4, was positioned on the top of the phantom, and the distance between the source and that collimator was adjusted to be 295 mm.

**Figure 4:**
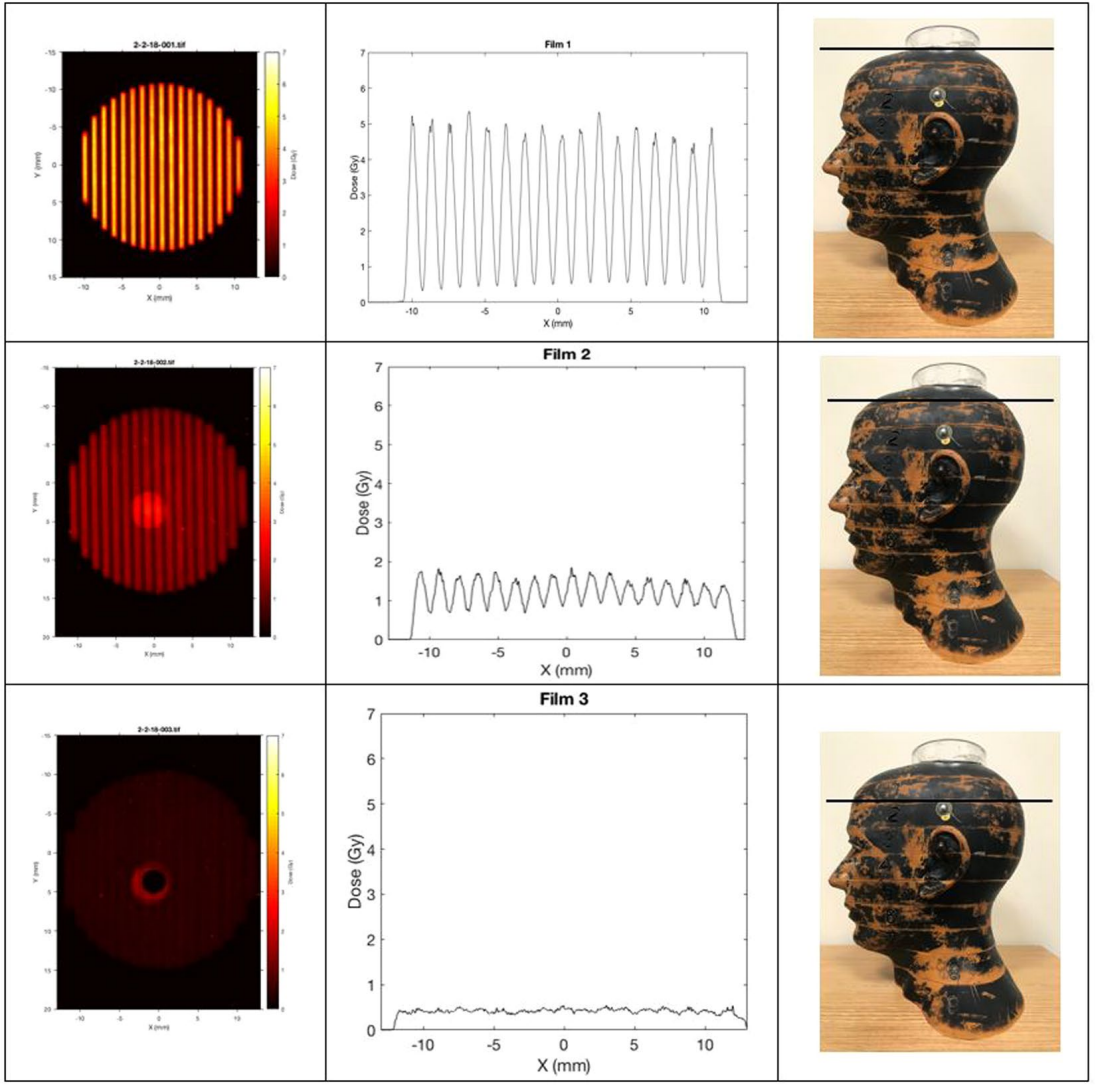
Cross sectional film exposure through the Alderson phantom. The irradiations were done vertically down from above the phantom and the multislit collimator positioned just above the phantom at 295 mm from the source (Fig. 3). One film was positioned above the phantom, one below the first slice, and a third underneath the second slice. The dose merging out of the primary collimator was 16 Gy, and the dose reaching the multislit collimator on the phantom’s top surface was slightly below 6 Gy. For each exposure, the left, middle, and right columns show the cross sectional film exposure, the array profile, and the height of the phantom at which the exposure was made, respectively.

In the first set of measurements, we used a humanoid phantom, namely the Alderson Radiation Therapy Head and Neck Phantom, to measure the cross sectional patterns of a two-dimensional array of x-ray minibeams passing vertically down from the top of the head through a collimator (Fig. 3). Film measurements were carried out on the top of the head for the non-attenuated beam, and then in between the upper four one-inch slices of the phantom. These cross-sectional patterns vividly demonstrate the gradual merging and attenuation of the minibeams as they go through the skull and the head tissues. The measurements involved the placement of films in between consecutive one-inch slices of the phantom. The results were obtained using a diverging collimator with a beam thickness of 0.34 mm and beam spacing of 1.12 mm on-center. The source-to-collimator distance was 295 mm. The results are presented in Fig. 4.

In this figure, the first column presents the cross sectional images as a function of the depth in the phantom, the second column shows the profiles of these cross sections, and the third column shows the depth in the phantom. The results in the second column, i.e. the array profiles, are presented in the absolute radiation dose in units of Gy. The depth measurements were continued until complete merging of the minibeams in the array occurred.

According to the results presented in Fig. 4, full beam merging occurs approximately between phantom slices 1 and 2, i.e. 2.5–5.0 cm from the top of the phantom. The phantom was positioned 2.5 cm below the multislit collimator making the beam merging about 5.0–7.5 cm from the multislit collimator. Furthermore, according to these results, the beam attenuation through the material from which the phantom is made has a half-value layer (HVL) of about 1.55 cm. This is considering the following approximate peak doses of 5.4, 1.7, and 0.5 Gy for the depths of 0, 2.5, and 5 cm from the top of the phantom.

The above discussion indicates the peak doses. The corresponding valley doses in the above images are approximately 0.42, 0.72, and 0.5 Gy for the above three depths, respectively. We would like to indicate that in general, and particularly in our measurements appearing in Fig. 4, the peak doses have no biological significance, while the valley doses may or may not have such significance. Specifically, in these examples, the value of the valley dose is insignificant in the first row, starts to become significant in the second row, and by the third row represents the only significant value. The above points emphasize the fact that in general in OXM, the significance of the dose lies mostly in the valley dose.

#### A2. Film Dosimetry

**Figure 5:**
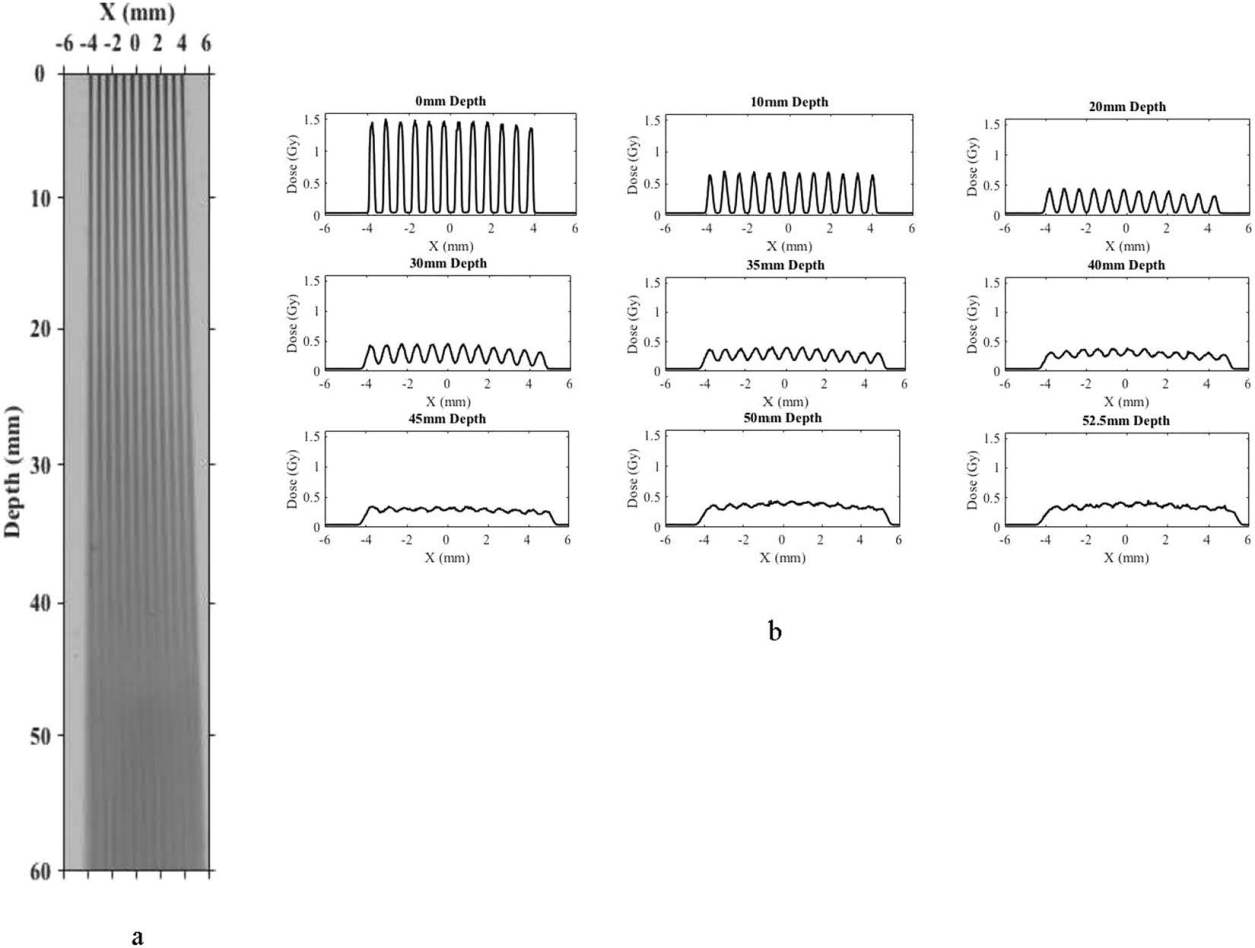
(**a**) Exposure of a chromographic film to an array of 0.3-mm 320-kVp x-ray minibeams spaced 1.0 mm on-center. The merging point is 3.1 cm from the collimator. (**b**) Lateral profiles of the film image of (**a**), made for nine depth points ranging from 0 mm (entrance to the film) to 80 mm, which is beyond the merging point of the arrays, which is at about 31 mm.

The profile results presented in Fig. 5a,b are significant in several ways. First, they show that within a 20.0 mm increase in the tissue depth the array changes from a clear microbeam with tissue sparing effect (22.5 mm depth) to a clear solid beam with a therapeutic property (42.5 mm). This means that major consideration in the design of the OXM’s clinical treatment planning would be to design the minibeams’ merging depth so that it spares as much proximal tissues as possible while producing therapeutic dose at the target site. Of course, the design will have to be drastically revised if tumor-dose-enhancing contrast agents are used.

The physical process involved is an increase in the radiation leakage between the minibeams, called “the valley dose”, as they get close to each other. That dose is produced by Compton scattering and by the finite ranges of photoelectrons and Compton electrons in tissue. The tissue depth at which the minibeams merge for a given source spot size can be adjusted by adjusting the source-to-collimator distance or the minibeam spacing.

### Part B. Monte Carlo Simulation Results

First, as indicated above, Fig. 2 includes the Monte Carlo simulations of the depth-dose distribution of physical absorbed dose on the unsegmented 300-kVp x-rays as well as the valley dose produced by a minibeam array in water. The minibeam array was assumed to be made of 0.3-mm thick planar beams spaced 1.0 mm on-center. Also, as mentioned above, our earlier studies indicate that the tissue damage produced by the microbeam/minibeam arrays is nearly entirely produced by the valley dose and very little by the peak dose^10,12^. The valley dose curve presented in Fig. 2 to a certain extent represents the biological dose.

**Figure 6:**
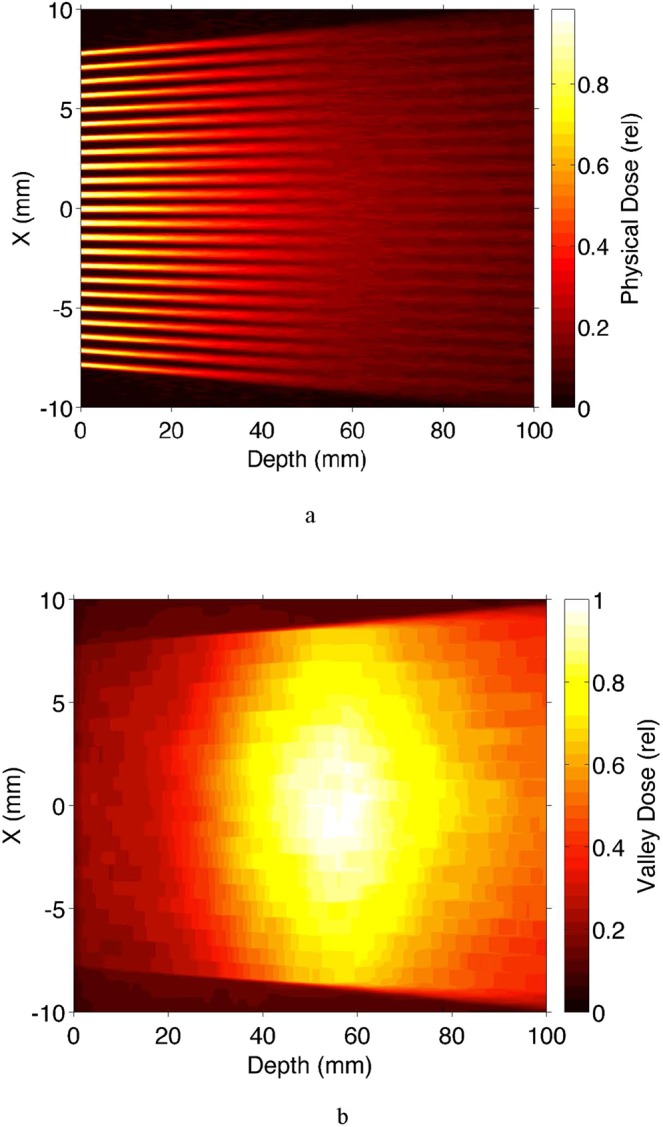
Monte Carlo simulations showing the top view of (**a**) the overall physical absorbed dose, and (**b**) the valley dose, produced in water by an array of 0.3 mm beams spaced 1 mm on center in 10-cm water.

Second, the same Monte Carlo was used to produce the top view of the absorbed dose and the corresponding valley dose, respectively, for the same minibeams array passing through water (Fig. 6a,b). The results presented in Fig. 6a indicate a microbeams’ merging depth of approximately 55 mm in the tissue for this geometry. The valley dose distribution shows a peak at approximately 55-mm depth. We note that the results from Fig. 2 show that the OXM’s physical skin-entrance valley dose is about half of that of the physical skin-entrance absorbed dose from 6-MV x-rays. Furthermore, the entire height of the curve presenting the minibeams’ valley-dose is lower than that of the curve for the 6 MV x-rays. These values highlight the great potential of OXM to treat deep brain targets with little or no damage to the shallow tissues, compared with 6 MV x-rays.

#### OXM reduces the dose distal to the target compared to MV X-rays

As made clear from the results of Fig. 2, another advantage of OXM over MV x-rays in the treatment of brain tumors is its smaller dose distal to the target due to the rapid attenuation of the lower energy photons. Our quantitative analysis in Fig. 2 indicates that the dose ~3 cm distal to a 2-cm target in a 14-cm water phantom from 6-MV x-rays relates to that from OXM as ~1.7:1.0. Although the use of intensity-modulated radiation therapy (IMRT) and VMAT with 6 MV photons ameliorates the effect of the distal dose on the contralateral cerebral cortex, as described below, OXM can also be implemented in IMRT or VMAT when using arc scan.

#### OXM produces sharper lateral dose falloff than MV x-rays

**Figure 7:**
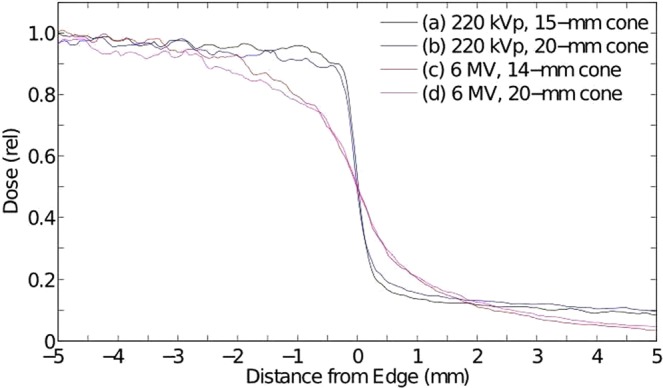
Lateral dose profiles of a 220-kVp and a 6-MV beam measured on a film stacked between two 4-cm plastic plates; it demonstrates the very large difference between the lateral dose-falloffs of these two beams, namely 80%-to-20% dose falloffs of ~0.4 mm and 2.2 mm, for the two beams, respectively.

As indicated above, the relatively low energy of orthovoltage x-rays, mainly in the 80-to-320-keV range, makes their mode of interaction with tissues largely photoelectric. This is as opposed to MV x-rays, mainly 500 to 2,000 keV, that attenuate primarily by Compton scattering. One consequence of this effect is that their lateral dose falloff would be significantly less sharp than that of the orthovoltage x-rays. We quantified this difference by measuring the dose falloffs of solid-beam 220-kVp and 6-MV x-rays on the same radiochromic films positioned between two 4-cm thick plastic phantom slabs. Specifically, the 220-kVp beams were collimated with either 15- or 20-mm diameter cone collimators, while the 6-MV beams were collimated with 14 or 20-mm cone collimators. The results, presented in Fig. 7, indicate that the 80%-to-20% dose falloff values were 0.40 mm and 2.2 mm for the orthovoltage and MV beams, respectively; this presents a 5.5:1.0 sharper dose falloff for the 220-kVp beam. One significant aspect of having such a sharp dose falloff is that the target’s volume to be irradiated will be smaller, just large enough to cover the tissues within their sharp dose falloff walls. On the other hand, the curves from these two energies have another difference, and that is the height of their tails when departing from the edge of the target by more than 2 cm. Specifically, the tail of the 220-kVp exposures are higher than those of the 6-MV x-rays. This is because the x-ray scattering produced by the 220-kVp x-rays, although quite small in intensity, is mostly of Thompson scattering nature, which has a large lateral component. This is as opposed to Compton Scattering from 6-MV x-rays which is mostly peaked forward with little side bands. This means that although the 220-kVp x-rays allow us to confine much of the dose to the geometrical shape of the target, they also have a detrimental effect of producing a small long tail of radiation depth-dose coming out sideways from the target. This combination of effects makes the comparison of the integral dose to the non-targeted tissues from OXM and MV x-rays difficult.

#### OXM can be implemented in arc-scan, combined with IMRT and/or VMAT

**Figure 8:**
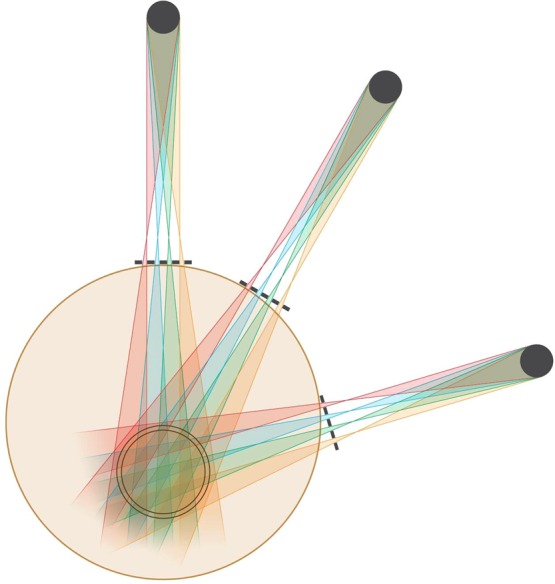
OXM’s exposures of a target from three shallow angles.

**Figure 9:**
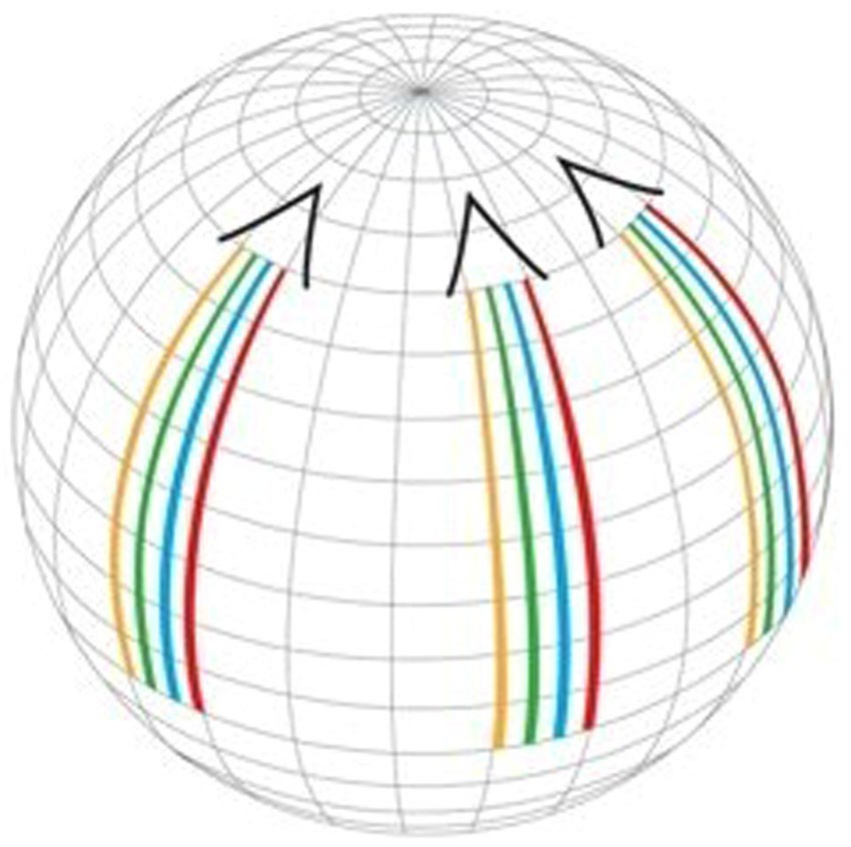
OXM’s implementation of three arc scans corresponding to the three exposure angles of Fig. 8.

To illustrate the concept, we first show in Fig. 8 a pattern of three OXM arrays aimed at a target from relatively shallow angles. In each of these three exposures the source can be scanned around the target in a continuous or stepwise arc geometry. This arc-scan concept is demonstrated in Fig. 9. The main requirement for this arc-scan to occur without mixing the adjacent minibeam planes is that the scan should be parallel to the planes of the individual minibeams inside the array. Also, the array should be rather small in the number of minibeams, so that all minibeams can be aimed at the target without the need to focus the array at a single target point. In this way, scanning along the arc keeps the minibeams individually separated along the way. To avoid the minibeams to collide with those aimed at the target from the opposite direction the arc’s rotation should be smaller than 180°. Several arcs can be used for the same treatment (Fig. 9).

We note that the arc scans can be implemented in volumetric modulated arc therapy (VMAT), the same way the clinical arc therapy is done^17^. Both methods use a multileaf collimator (MLC) for adjusting the shape and the size of the radiation field. As for the difference between them, unlike conventional IMRT treatments during which the machine exposes the patients in the step-and-shoot fashion as it rotates around the patient, VMAT dynamically delivers the dose as the gantry arcs across the patient. In this sense, implementation of OXM with VMAT will require dynamic adjustment of the source-to-collimator distance along the arc to produce the optimal beam-merging tissue-depths. Although this would make the method’s treatment planning more challenging, it is within the reach of the current technology. In both VMAT and IMRT, the MLC will be positioned immediately behind the multislit collimator with the latter being positioned very close to the skin. Furthermore, the slit optimization and the adjustments of the source-to-collimator distance to optimize the merging depth of the minibeams might have to be revised during the course of the treatment to account for possible tumor shrinkage and/or changes in the patient’s body weight. As indicated in the Methods and Experiments section below, this can be done by dynamically varying the distance between the source and the multislit collimator. Finally, as indicated in Methods and Experiments below, the minibeams’ merging point can be varied by changing the inter-beam spacing of the collimator; however, that cannot be done in a dynamic way at this point in time. As for the target dose homogeneity, we expect that adequately designed dose-painting parameters will provide a quite uniform target dose. In this regard, we also note that the method’s forgiveness in terms of the height of the incident minibeam array dose would provide the user an open hand to adjust the incident dose and a possible non-uniform incident minibeam dose through the exposure area to optimize the target dose.

#### OXM is ideal for tumor-dose enhancement with contrast agents

As indicated above the OXM’s low beam energies makes the photoelectric effect to be its main mode of interaction with tissues. This makes OXM ideal for tumor dose enhancement with any high Z contrast agent^18^. These may include iodine, xenon, barium, gadolinium, and gold. As an example, iodine’s attenuation coefficient at 80 keV is 3.5 cm^2^/g and at 1 MeV is 0.058 cm^2^/g, i.e., 60-fold greater. This allows contrast-enhanced radiotherapy (CERT) at a small fraction of the dose necessary for conventional RT.

## Discussion

The tissue-tolerance of the minibeam arrays can be quantified approximately using specific examples in which x-ray minibeam and x-ray solid beam exposures were carried out in similar experiments in the same CNS model. As one such example we compare the synchrotron x-ray minibeam studies of Dilmanian *et al*.^5^ and the solid beam orthovoltage x-ray studies of Calvo *et al*.^19^, both carried out in the rat brain. In the former study, rats irradiated over nearly their entire brain with 0.68 mm planar minibeams spaced 1.36 mm on-center at 170 Gy dose not only displayed no behavioral changes over the 7-month observation period, but also gained weight normally^5^. In comparison, local 22.5-Gy solid beam irradiation of the rat brain led to “histological evidence for the development of necrosis in the white matter after a latent period of >26 weeks”^19^. This puts the dose-tolerance advantage of 0.68-mm minibeams to that of solid beams over 7-fold. However, as discussed below that factor can be much larger than 7-fold for narrower beam thicknesses and/or larger relative beam-spacing values.

The following describes the method of calculating the physical absorbed dose in the incident minibeams for producing a certain biological dose (valley dose) in the target for a given beam spacing value. For this purpose, we use the results presented in Fig. 2. First, the height of the dashed red curve in Fig. 2 was determined from the Monte Carlo simulations. Next, using the height of the dashed red curve in Fig. 2, we see the physical absorbed dose of the non-segmented orthovoltage beam presented as the black curve. However, connecting the absolute height of the black curve to the absolute height of the minibeam entrance dose, we have to correct for the dose dilution factor introduced by the use of minibeams. This dose dilution factor is 10/3 for using 0.3 mm minibeams spaced 1.0 mm on center. Finally, we have to correct for attenuation of the beam in the subject, which could also be given by Monte Carlo simulations.

**Figure 10:**
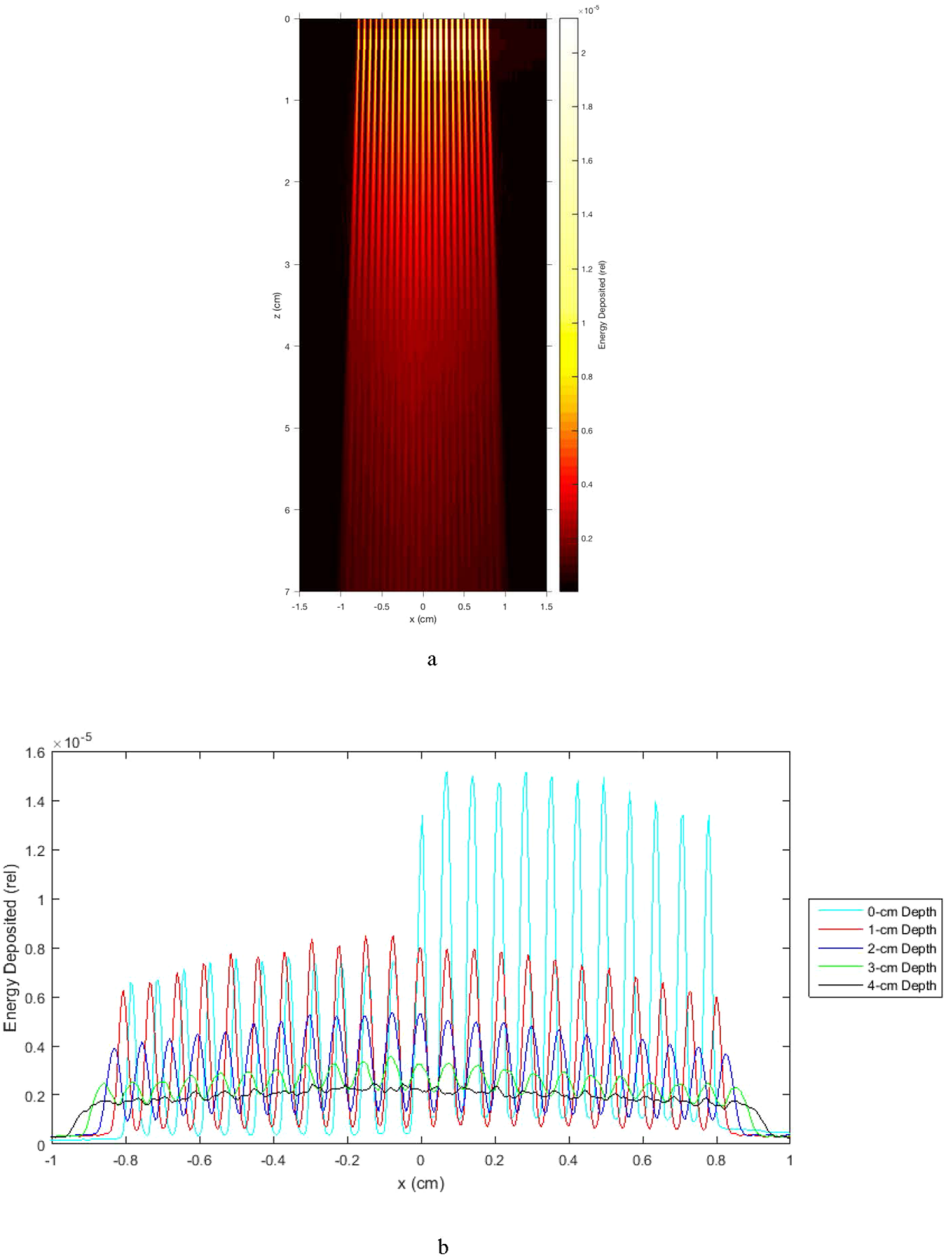
Effect of cortical bone on the OXM merge depth in water. (**a**) Shows Monte Carlo simulations of a heatmap of energy deposition by 320 kVp (3.8-mm Cu-HVL) xray minibeams, 300-micron, spaced 700 microns on center, 5-mm-thick tungsten multislit collimator with parallel blades, with a simulated 5-mm slab of cortical bone (density = 1.85) at x > 0 and 0 < z < 0.5 cm. Increased energy deposition is seen in the bone region and a slightly decreased intensity of minibeams is seen in water at x > 0 and z > 0.5 cm, due to attenuation in the bone. Nevertheless, this attenuation led to minimal effect on the merge depth of the minibeams, which occurs at approximately 4-cm depth in water. (**b**) Shows the horizontal profiles of (**a**) at depth intervals of 0, 1, 2, 3, and 4-cm for the entire horizontal width of the array. As indicated in the description of (**a**), the profiles vividly demonstrate that a 5-mm bone has a large effect on the attenuation of x-rays, while it has almost no effect on the merging depth of the minibeam array. The latter is clear from the fact that the profile at the 4-cm depth is completely flat from left to right.

**Figure 11:**
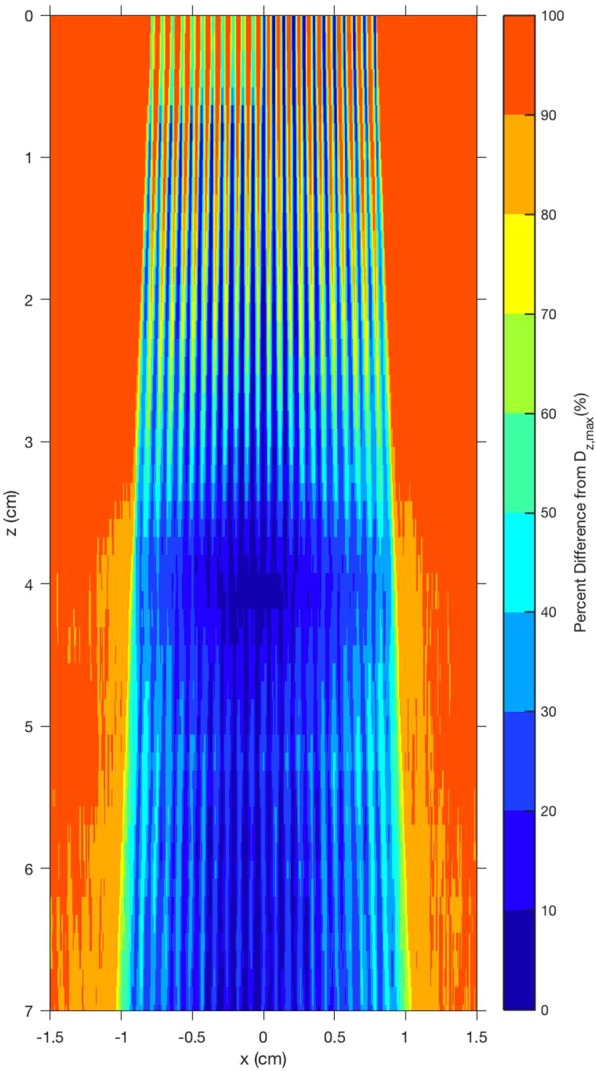
The local peak-to-valley ratios are quantified as the percent difference between the peak of the local energy deposition and the minimum value, called valley, of the energy deposition for a given depth in the subject. Lower percent difference indicates a region with less relative variation in peak to valley values. The figure reveals a homogenous merge region near 4-cm depth, which is approximately symmetric despite the presence of cortical bone at the surface, which blocked half of the beam at x < 0. As a subtle point, being revealed in both Fig. 10a,b, the measurements would have benefited from the use of a diverging multislit collimator. Such a use would have produced a more uniform lateral dose distribution, producing a “flat” merge depth. The need for the diverging multislit collimator depends on several parameters including the source spot size, the source-to-collimator distance, and the collimator thickness for a given beam thickness and beam spacing.

Our Monte Carlo investigations on the role of cortical bone and a varying skull thickness (Figs 10a,b and 11) revealed that even a sharp discontinuity in skull thickness led to minimal (<10%) effect on the underlying dose distribution in the brain. Thus, we infer that the curvature and variation in skull thickness will not likely prove to be a significant obstacle for translation of the method to human therapy.

The cross sectional beam attenuation results presented above indicate a dose HVL of about 1.55 cm in a practical brain tumor phantom. Although this means sharp dose falloff with tissue depth, resulting in a larger proximal dose being required, it should still allow the clinical implementation of OXM because of its substantial tissue-sparing of the minibeams in the shallow tissues. In other words, the skin, muscle, skull, and the cerebral cortex are all expected to tolerate the incident minibeam doses required in the clinical configurations. The tissue-sparing of the clinical minibeam arrays should be even larger than that because it will probably use 0.3 mm (and not 0.68 mm) minibeams spaced about 1.0 mm on-center, i.e., narrower and farther-spaced minibeams than those used in the above example.

**Figure 12:**
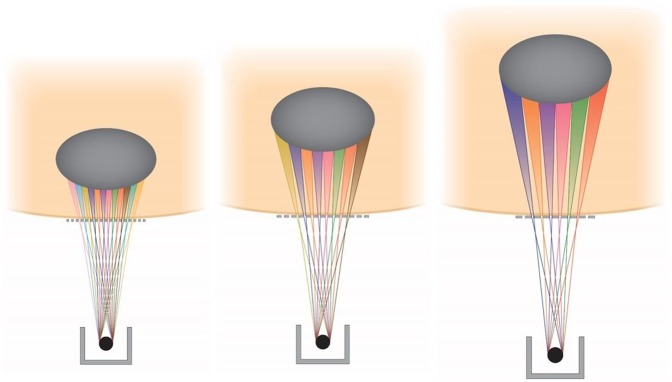
Adjusting the tissue depth at which the minibeams merge by varying the spacing between the minibeams.

Yet another method to adjust the merging depth is to use multislit collimators with different values of beam spacing. This effect is demonstrated in Fig. 12, in which the merging depth is presented for three different values in beam spacing, all using the same source-to-collimator distance.

For the following reasons the OXM’s largest clinical significance would be its sparing of the cerebral cortex. First, there are indications that conventional radiation therapy can produce significant radiation damage to the cerebral cortex, particularly in children^20,21^. Specifically, Karunamuni *et al*.^20^ showed a thinning of the cortex up to 0.3 mm for the highest dose (60 Gy). To put this into context, the annual thinning of the cortex is 0.1 mm for an Alzheimer’s patient^22^ and only 0.02 mm in the control aging population^23^. It is known that portions of the cortex are critical in neurocognitive functions such as information processing and memory. Consequently, thinning of the cortex could negatively influence these functions.

Second, the cerebral cortex is the brain’s second structure, besides the hippocampus, for producing neural stem cells (NSCs)^24,25^. However, while the NSCs produced by the hippocampus mostly differentiate into neurons, those produced in the cerebral cortex are mostly progenitor glial cells that undergo gliogenesis to produce oligodendrocytes and astrocytes^25,26^, although some also undergo neurogenesis to develop into neurons^25^. Furthermore, although both of these processes are most prevalent in the developing brain, they continue to some extent also at higher ages^26^. Both of these processes are major cornerstones of the development of the brain’s function and cognitive performance. Therefore, their possible disturbance by radiation damage leads to impairment of core cognitive functions such as learning, attention, working memory, and the speed of information processing^24–26^. As for the radiosensitivity of the NSCs, while mature neurons are not affected directly by radiation because they are post-mitotic, the brain’s actively dividing NSCs are highly sensitive to radiation and can be largely wiped out even at very small doses^27^.

Finally, another major path through which conventional radiation therapy damages the brain is the brain’s microvasculature. In this regard, it has been shown that conventional radiation therapy can damage the entire vasculature by damaging the vascular endothelial barrier, also known as the blood-brain-barrier. Specifically, Rodgers *et al*.^25^ showed increased permeability of the barrier due to radiation in the rats’ brains through electron microscopy. This means that toxic substances and inflammatory cells and molecules can pass through uncontrolled, disrupting the microenvironment in the brain.

In summary, the above material explains the very large radiosensitivity of the cerebral cortex, particularly in children.

We note that none of the current radiation therapy methods spares the cortex as much as OXM potentially can. First, MV x-rays spare only the skin and some of the skull (see Fig. 2). Second, although Gamma Knife does spare the cortex for treating small targets, for large target treatments it produces a significant background dose over the entire brain including the cortex, similar to that seen with MV x-rays implemented in IMRT and VMAT geometries^17^.

The sequela of brain radiotherapy with solid beams in adults includes early development of dementia, clinical depression, and even speech disturbances. Furthermore, whole brain radiation commonly used for treating metastases leads to damage of the white matter as well^28^. Such irradiations often cause irreversible damage to myelin that in turn leads to development of leukoencephalopathy^28^.

It should be noted that the degree of cognitive decline in children has been found to directly correlate with high integral brain dose^29^. From the detailed information given about OXM’s dose-saving to the tissues positioned proximal, lateral, and distal to the target, we expect that it will also excel in this category. This is in spite of a small long-range lateral dose that our phantom studies showed.

A most important attribute of any radiation therapy method is the tightness of the dose to the target it produces compared to that of the conventional method(s). In this regard, OXM’s value is clear. Its proximal tissue sparing, smaller distal dose, and sharp lateral dose falloff should produce a very well-defined dose to the target compared to that of MV x-rays. This makes OXM ideal for treating not only brain tumors but also any other tumor that does not move during the breathing cycle and will benefit from a target dose with sharp dose falloff; such tumors will include head-and-neck tumors as well as tumors located on or near the spinal cord. Furthermore, the dose tightness to the target would be particularly beneficial for treating rather large targets of focal epilepsy; treating such targets with the current radiosurgery methods often produces considerable amounts of edema, intracranial pressure, and local brain damage^30^. Finally, the method could be applied to shallow tumors of the chest or abdomen as long as the tissues proximal to the target can be adequately immobilized. This makes the method more applicable worldwide.

The tissue-sparing of x-ray microbeams and minibeams are thought to be based on two biological mechanisms, namely the dose–volume effect^31,32^ as well as a “prompt, microscopic biological repair” effect^9–12^. The first effect explains the higher dose tolerance of small targets that constitute a small portion of the entire organ^31^ and is the basis for stereotactic radiosurgery^33^ as well as Grid Therapy^33^. The second effect, which occurs only in exposures with sub-millimeter beams, is caused mostly by the rapid repair of the capillary blood vessels from radiation damage by microbeams and minibeams^9–12^. Specifically, studies on the chicken embryo^9^ and the mouse brain^11^ show an extremely rapid (within 12 hours) recovery of the capillary blood vessels following exposure to microbeams of up to 2 to 3 hundred Gy. The subject is summarized in ref.^12^.

The above discussion of the microbeams’ “prompt, microscopic biological repair” effect indicates that OXM is categorically different from Grid Therapy^33^. This is because of the latter’s large hole-sizes, up to 1-cm or larger. As a result, Grid-Therapy’s skin sparing effect solely comes from the dose-volume effect. On the other hand, the tissue-sparing effect of OXM originates from both the dose-volume effect and the prompt, microscopic biological repair effect.

In summary, our physical feasibility studies, focused on the treatment of benign and malignant brain structures, indicate that OXM significantly reduces the dose to non-targeted brain matter. This is achieved by (a) sparing the proximal tissues or significantly reducing the radiation damage to them, (b) reducing the dose to tissues positioned distal to the target, and (c) reducing the dose to tissues positioned immediately lateral to the target by virtue of producing a sharper lateral dose falloff. These dose savings should translate into significant sparing of the cortex and other critical brain structures, and thus lead to a significant reduction in cognitive deficits. In particular, saving the cortex can prevent the disrupttion of pediatric cerebral cortical gliogenesis^25^, a process producing progenitor cells that later differentiate into new oligodendrocytes and astrocytes. We expect to save much of the frontal cerebral cortex and significantly reduce the dose to the contralateral cortex from each exposure angle.

We note that in principle OXM’s sparing of the proximal tissues requires that the tissues will not move during the irradiation, as such movement can smear the minibeams’ dose, producing a continuous beam that would be damaging. This makes the patient immobilization more critical for OXM. The risk will depend on the array’s minibeam thickness and spacing, as well as on the magnitude of the tissue excursion. In that regard, the cardiovascular pulsation of the brain should be tolerable when using arrays of 0.3 mm beams with 1.0 mm spacing on-center because the amplitude of these pulsations should be less than 0.7 mm^34^. The risk for such beam smearing can further be reduced by increasing the beam spacing on-center beyond 1.0 mm for the same 0.3-mm beams. However, the use of larger beam spacing will require the use of higher incident dose in each minibeam. This, in turn, would reduce the effective dose rate produced at the target. In that regard, we expect that an array of 0.3 mm minibeams spaced 1.0 mm on-center will be tolerated by the skin at in-beam doses of more than 170 Gy, which was the dose tolerance to 0.68-mm minibeams with 1.36-mm beam spacing on-center^5^. The use of a thermoplastic-based mask system to immobilize the patient’s head for OXM administration should be adequate, and the beam’s attenuation in the mask can be corrected for.

Although for the last 60 years the subject of orthovoltage x-ray machines has been nearly completely outside the fields of radiation oncology and radiosurgery except for certain small applications, OXM’s unique dosimetric attributes, coupled with OXM’s innovative treatment delivery strategies with or without the use of tumor-dose-enhancing contrast agents, should allow resurrection and repurposing of orthovoltage x-ray machines for a full range of clinical treatment scenarios.

Although not directly related to OXM, synchrotron-generated minibeams of 52-µm thickness have been used at the ESRF in an interlacing geometry from four 90° angles to produce a 200-µm–thick solid beam at a small target in the rat brain^15^. We also note that the research group at University of North Carolina has been producing ~0.5-mm thick x-ray microbeams (or, as we call them, minibeams) using carbon nanotube field emission^35^. However, there is no indication that the authors will use their beams in a merging geometry as used in OXM.

Given OXM’s sparing of the tissues proximal to the target and its lower doses distal and lateral to the target imply that the dose produced by OXM is significantly more confined to the target than that produced by MV x-rays.

Finally, OXM has a practical advantage over the MV methods because its construction and room shielding are less demanding and its treatment planning would also be easier.

## Methods and Experiments

### Phantom studies using cross sectional array exposures of films

The studies used chromographic films, namely Gafchromic EBT, for dosimetric measurements. The film exposures were then scanned without using energy-dependent film sensitivity correction. The F2 filter used was made of 1.5 mm Al, 0.25 mm Cu, and 0.75 mm Sn. All irradiations were performed on a 250-kVp orthovoltage source at PXI using 16 Gy incident minibeam dose. A diverging multislit collimator made of 5 mm thick tungsten was used. The collimator’s focal depth was 295 mm. Its beam thickness was 0.34 mm and its beam spacing was 1.12 mm on-center.

### Monte Carlo simulation of physical absorbed dose and the biological dose produced by a minibeam array

Monte Carlo simulations of OXM were carried out using MCNPX version 2.7 (Los Alamos National Laboratory) using the default physics models for photon and electron transport, including photoelectric absorption, Compton scattering, secondary electron transport, and production of Bremsstrahlung photons, and a lower energy transport cutoff of 10 keV. Orthovoltage photons were transported from a disk source with a radius of 3.4 mm positioned 29.5 cm upstream in air of a 20 × 20 × 20 cm^3^ water phantom. Photon energies were sampled from an existing spectrum for an MXR 321 tube at 320 kVp with a 3.8-mm Cu HVL. Photon directions were isotropic but restricted to a forward-directed cone that contained the water phantom, similar to the effect of a shielded x-ray tube (virtual primary collimator). Absorbed dose was tallied in rectangular voxels with 50 × 50 × 1000 μm^3^ dimensions in x, y, and z, respectively, with z parallel to the beam’s central axis). A total of 5 billion photon histories were tracked for each simulation. For simulations with OXM, a 5-mm-thick tungsten multislit collimator was modeled immediately upstream of the water surface, having an array of 23 parallel apertures of 300 μm-width, spaced 700 μm apart on-center, and 2-cm aperture length.

To simulate a solid orthovoltage beam (conventional orthovoltage therapy), the MCNPX settings were identical to those for minibeams except (1) the source was positioned 50-cm upstream of the water phantom and (2) a 10 × 10 cm^2^ open aperture was modeled instead of the multi-slit apertures. For comparison with 6 MV x-rays, a measured 6-MV depth dose curve (Varian TrueBeam accelerator, 10 × 10 cm^2^ field size, 100-cm source-to-surface distance (SSD), from the University of Maryland School of Medicine (Baltimore, MD)) was included.

### Prediction of the biologic dose produced by OXM

To estimate the biologic effect of OXM, we considered that, to a first order, the most important physical quantity that can be used to predict the biologic tissue damage after minibeam exposure is the valley dose. Whereas cells exposed to minibeam peak doses may be completely sterilized, the cells surviving in a nearby valley are expected to migrate and repopulate to repair the organ. This mechanism relies on the assumption of a maximum distance over which such repair can occur, which we estimate to be 350 µm based on the experimental data^5^ showing tolerance of CNS tissue to 700-µm-width minibeams. In that data, the maximum distance between a cell in the middle of a minibeam peak and the nearest valley would thus be approximately 350 µm. We implemented these assumptions in a computer model to predict the biologic dose by analyzing Monte-Carlo-simulated absorbed dose distributions. For each dose point, the computer model also searched for the minimum dose in a local region with a radius of 350 µm about that point, i.e., the dose in the nearest valley. Using this simple model, we compared the predicted biologic dose for orthovoltage minibeams against that of orthovoltage broad-beams and megavoltage broad-beams. The predicted biologic dose for broad beams was assumed to be identical to their physical dose.

### Methods of adjusting the merging depth of the minibeams in the subject

As indicated above the merging depth of the minibeams can be adjusted in two ways. First, the adjustment is achieved by varying the distance between the source and the multislit collimator (Fig. 1). As the source gets closer to the collimator, the merging depth of the minibeams in the subject is decreased, i.e., the minibeams merge with their neighbors at a shallower depth in the subject. Alternatively, the minibeams’ merging depth could be adjusted by varying the spacing between the minibeams (Fig. 12).

### OXM allows fine tuning of the minibeams’ merging depth in tissues

Figure 5a shows a chromographic film exposed to an array of minibeams 0.3 mm thick, spaced 1.0 mm on center. It was produced by a 320-kVp PXI machine called XRAD-320. The source size was 6.0 mm. The study used a parallel multislit collimator, positioned 315 mm from the source. The film presented here was positioned directly below it. The width of the array at the entrance to the film was 8.0 mm. The film was scanned using an Epson Expression 10000XL scanner with 3,200 dpi resolution. Pixel profiles were then converted to dose using the scanner’s response curve and plotted versus distance along the width of the beam array at nine different distances from the entrance of the array to the film. The results are presented in Fig. 5b.

### Geometrical considerations in the clinical implementation of OXM

The information presented in the frames of Fig. 5b on the minibeams’ gradual merging should be put in the context of treatment planning administered from different angles. The merging process will become more involved when the subject is treated with arrays aimed at the target at fairly shallow angles, as presented in Fig. 8. In particular, if the collision between the arrays occurs before the minibeams in each array merge to produce a solid beam, partially-overlapping arrays will result outside the target with a complicated dose distribution called “star artifacts.” This effect, which can be simulated for patient treatment, should be taken into account in treatment planning.

### Dose-rate considerations in the clinical implementation of OXM

As indicate above, when XRAD-320 operates at its maximum current of 9 mA with beam filtration producing a 3.9 mm Cu HVL beam, it provides a dose rate of 10 Gy/minute at a 10-cm distance from the source. We argue that this beam yield is adequate for the clinical implementation of OXM for treating brain tumors with or without additional beam filtration. This is because the very high tolerance of all tissue types to minibeams makes the method very forgiving in terms of the entrance-dose allowance. In fact, our preliminary studies have indicated that the minipig skin, skull, and brain tolerate 275-Gy in-beam incident minibeams with no hair loss or skin damage at any time after the exposure.
